# Directional and Eye-Tracking Light Field Display with Efficient Rendering and Illumination

**DOI:** 10.3390/mi14071465

**Published:** 2023-07-21

**Authors:** Guangyong Zhang, Yong He, Haowen Liang, Xuehao Chen, Dongyan Deng, Jianying Zhou

**Affiliations:** 1 School of Physics, Sun Yat-sen University, No. 135 Xingang-Xi Road, Guangzhou 510275, China; zhanggy37@mail2.sysu.edu.cn (G.Z.); lianghw26@mail.sysu.edu.cn (H.L.); chenxh87@mail2.sysu.edu.cn (X.C.); 2 Guangzhou Midstereo Co., Ltd., No. 135 Xingang-Xi Road, Guangzhou 510275, China; dengdy@midstereo.com

**Keywords:** directional backlight, super multi-view, light field display

## Abstract

Current efforts with light field displays are mainly concentrated on the widest possible viewing angle, while a single viewer only needs to view the display in a specific viewing direction. To make the light field display a practical practice, a super multi-view light field display is proposed to compress the information in the viewing zone of a single user by reducing the redundant viewpoints. A quasi-directional backlight is proposed, and a lenticular lens array is applied to achieve the restricted viewing zone. The eye-tracking technique is applied to extend the viewing area. Experimental results show that the proposed scheme can present a vivid 3D scene with smooth motion parallax. Only 16.7% conventional light field display data are required to achieve 3D display. Furthermore, an illumination power of 3.5 watt is sufficient to lighten a 31.5-inch light field display, which takes up 1.5% of the illumination power required for planar display of similar configuration.

## 1. Introduction

Three-dimensional scene perception without wearing glasses can offer convenience; hence, glasses-free 3D display has attracted great attention in recent years. Substantial studies have been conducted to realize glasses-free 3D display [1,2,3], among which, conventional autostereoscopic display simply sends parallax images to viewer’s eyes. The technique of autostereoscopic display is now quite mature, but the simple principle determines that the 3D impression is not natural because of the vergence-accommodation conflict [4,5]. Light field display is another kind of glasses-free 3D display which can optically redistribute the spatial information of a 3D scene. Since the scene is reconstructed by the light field, a clear 3D scene with correct geometric occlusion and smooth motion parallax can be observed by the viewer.

Light field display can be realized with various methods [6,7,8,9,10,11,12,13,14,15,16], among which multi-projection scheme is a common technique [17,18]. Smooth motion parallax and high definition can be achieved with high dense projectors, but considerable calibration efforts and complex system arrangement present technique difficulties. It is also a giant challenge to handle the massive data and control all projectors synchronously. Compressive light field display (or multi-layer tensor display) which consists of multiple liquid crystal displays (LCDs) is another 3D display technology [19,20]. The compact structure is among the advantages of compressive light field display, but the low brightness caused by multiple LCDs degrades the quality of 3D images. Furthermore, massive complex calculations are required to decompose the target light field information loaded on multiple LCDs. Integral display which is the inverse process of integral photography can provide natural 3D scenes with full color and full parallax by optically reconstructing light field of the 3D scene [21,22]. In addition, the structure of integral display which mainly includes an LCD panel and a lens array is comparatively simple, while the low resolution and massive data are the difficult issues to tackle. Multi-view display can be considered as a super multi-view (SMV) light field display if the viewpoints are dense enough [23]. The optical structure of integral display and SMV are similar despite the pixel arrangement in the image source being different. SMV light field display generates dense viewpoints at the optimal viewing distance (OVD) to provide smooth motion parallax, while the large number of viewpoints normally lead to massive data and low definition of 3D images.

All light field displays mentioned above can offer 3D scenes in a relatively wide range, and numerous studies have concentrated on the effort to achieve the widest possible viewing angle. The space-multiplexing technique is introduced to realize a 90-degree viewing angle [24], and a 120-degree perception is achieved using the time-multiplexing technique [25]. A tabletop light field display with FOV of 70° × 70° is also achieved [26]. The simultaneous wide viewing angle will require massive information. Furthermore, the wide viewing angle is at the cost of the reduced resolution. In the flat-panel (such as LCDs or LEDs)-based light field display, the pixels of the flat-panel are converted to the angular and spatial information for 3D images. Since the total space bandwidth product of the flat-panel are limited, large angular display in a wide range means that few pixels are converted to the designated direction, giving rise to low-definition 3D images. To tackle the trade-off among the spatial resolution, angular resolution, and viewing angle, a 3D display with spatially variant information density and a 160-degree horizontal viewing angle is proposed [27]. Stereoscopic experiences with smooth motion parallax are maintained at the central view, while the viewing angle is enlarged at the periphery view.

Some novel light field devices have also been proposed. According to human watching habits, a light field 3D display with space-variant resolution for non-uniform distribution of information and energy is proposed [28]. The pixel density of parallax images on various views is adjusted to achieve the variant spatial resolution. Meanwhile, the brightness of the parallax images also forms a non-uniform distribution. Light field display is also combined with head-mounted display, and a reverse pass-through virtual reality device is proposed to enable natural eye contact and other important non-verbal cues in interaction scenarios [29]. Additionally, 3D views of the user’s face and eyes are presented to outside viewers in a perspective-correct manner using a light field display.

**Figure 1:**
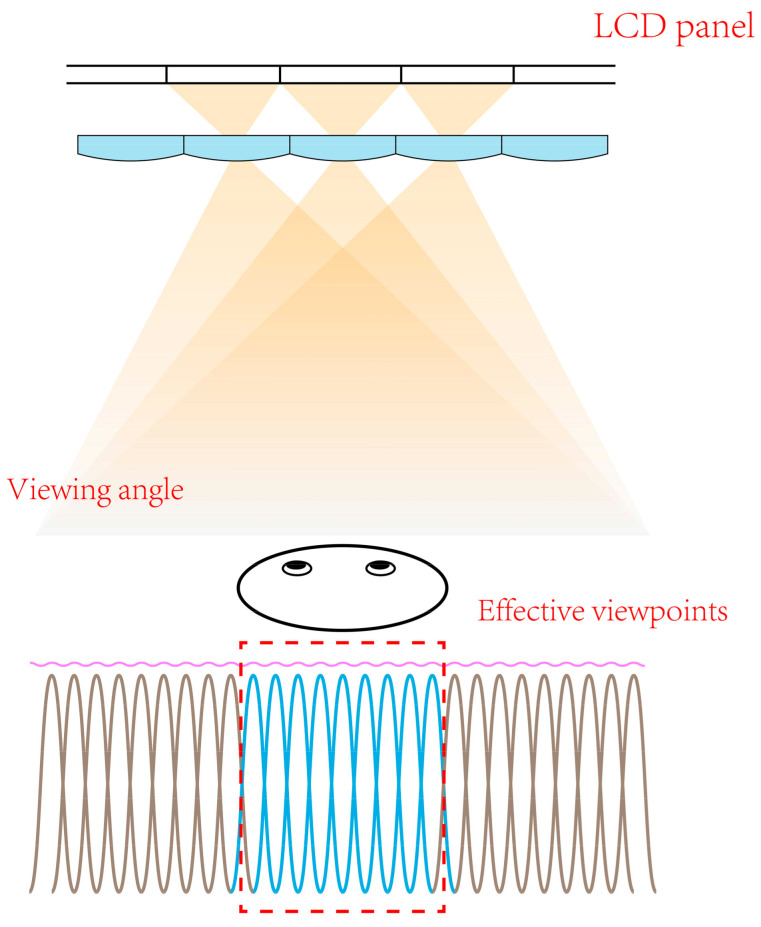
The effective viewpoints for a single viewer in a conventional light field display.

The wide viewing angle can provide 3D images for multi-viewers simultaneously, while a single viewer needs to view the display in a specific viewing position, as shown in Figure 1. Eye-tracking is another technique normally applied to a single user. Dorado et al. proposed a method using the eye-tracking technique to improve the viewing angle and the parallax provided by integral monitors [30]. The method demonstrates that it is possible to adapt in real time the displayed 3D image to the observer location. While lens array with a long focal length is applied to enhance the depth range of the integral display, eye-tracking can be further introduced to widen the viewing range [31,32]. For this purpose, Ueno presented an SMV near-eye display system based on a high-speed spatial light modulator with a 2D light source array [33], and 21 viewpoints with the density of 0.5 mm^−1^ were achieved with the time-multiplexing technique. Takaki demonstrated an SMV display with low resolution flat-panel [34], which could provide 3D images with viewpoints density of 0.38 mm^−1^ by providing only four viewpoints to each eye. However, the scheme decreases the horizontal width of the observation area, and the high crosstalk further deteriorates the display quality. Yang developed a crosstalk-suppressed dense multi-view light field display with micro-pinhole array [35], which could exhibit the 3D images with viewpoints density of 0.75 mm^−1^ and the crosstalk of less than 7%. However, applying the micro-pinhole array sacrifices the brightness of 3D images. Wang demonstrated a low-crosstalk SMV light field display with the vertically-collimated programmable directional backlight, and viewpoints dense up to 1 mm^−1^ were generated to provide natural depth cues and smooth motion parallax [36]. Nevertheless, the dense viewpoints were generated with a vertical diffuser, which blurs the 3D images.

Here, an SMV light field display is proposed to compress the information in the viewing zone of a single user to alleviate the information redundancy by reducing the redundant viewpoints. A quasi-directional backlight is proposed, and a lenticular lens array (LLA) with a long focal length is applied to specify the viewing zone. With the quasi-directional backlight and the LLA, the image transmission is consistent with the illumination direction. In total, 17 viewpoints with the density of 0.13 mm^−1^ are generated in a 5-degree range to ensure smooth motion parallax. Compared with the previous SMV light field display mentioned above, such a viewpoint density simultaneously reduces redundant information and ensures saltation-free parallax. Since the viewpoints are concentrated in the viewing zone of a single viewer, the viewing area is practically decreased. The eye-tracking technique is then introduced to extend the total observation range. By acquiring the position of the viewer in real-time, accommodating the LCD image and switching the directional backlight synchronously, vivid 3D images can be sent to the viewer retina precisely. The total viewing space is extended to 30 degrees.

With this configuration, the information utilization rate and the energy efficiency are improved substantially. Furthermore, the reserved small quantity of viewpoints reduces the requirements of information transmission bandwidth, hence providing a real-time render and display of light field information.

## 2. Principle

### 2.1. System Configuration

**Figure 2:**
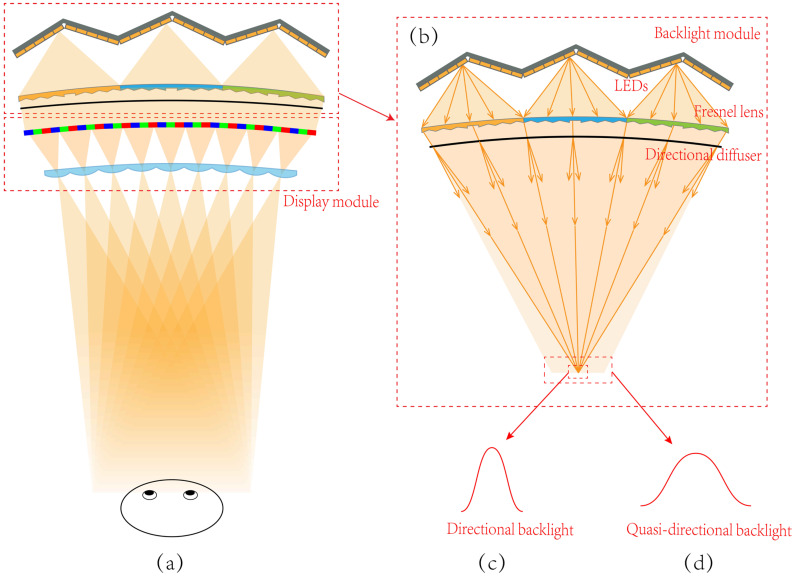
(**a**) The schematic diagram of the proposed light field system, (**b**) the quasi-directional backlight module, (**c**) light intensity distribution of directional backlight, and (**d**) light intensity distribution of quasi-directional backlight.

The proposed light field display contains the quasi-directional backlight module and the display module, as shown in Figure 2a. The quasi-directional backlight module is shown in Figure 2b, which consists of several units arranged side by side and distributed on a circular arc [37]. Each unit in the backlight module comprises a linear Fresnel lens (LFL) and a series of LEDs. The LEDs are arranged in a V-shape to fit the field curvature of LFL. In addition, a directional diffuser is utilized to achieve small angle diffusion in the horizontal direction. A 31.5-inch curved LCD panel with curvature radius at 1451 mm and with a resolution of 3840 × 2160 is adopted in the proposed light field display to load the encoded light field information. The LLA is placed in front of the curved LCD panel to control the light rays and generate viewpoints. With the LLA, the light field information loaded on the LCD can be decoded, and the viewpoints can be generated at the OVD (1451 mm away from the system). To ensure that the viewpoints can be generated precisely, the quasi-directional backlight, the curved LCD panel, and the LLA are placed on three concentric circles. In addition, the eye-tracking technique is further introduced to provide directional illumination for extended viewing area.

The quasi-directional backlight module is utilized as the illumination light source. The divergent light rays emitting from LEDs are collected and converged to the convergent light rays with well-defined direction by the LFL. By introducing the directional diffuser, the convergent light rays will diverge in a horizontal direction with a small angle. Although the directional information is interrupted by the diffuser, the degree of divergence in the horizontal direction is small to retain its directional property. After passing through the LCD, the encoded light field information loaded on the LCD is carried by the light rays. The LLA is placed in front of the curved LCD panel to collect the light rays with light field information and generate viewpoints at the OVD. With the LLA, the viewpoints are concentrated in a small viewing zone, and redundant viewpoints are reduced.

Each LED in the backlight module can be lighted up independently, and the direction of the convergent light rays are then different with different lighted LEDs. Hence, the position of the illumination area can be varied by switching the lighted LEDs. On the other hand, the information carried by the light rays can be updated through refreshing the LCD panel. Hence, the direction of information transmission can be regulated by synchronously controlling the directional backlight and refreshing the LCD panel with suitable pixel programming. With the directional backlight, the illumination is restricted within a 5-degree region. This range is considered to be suitable for a single viewer 1.45 m away from the LCD screen with the illumination width at about 13 cm, which is twice bigger than the nominal inter-ocular distance at 6–7 cm. At the same time, the information of the programmed LCD is concentrated in this region for the generated viewpoints. As a result, the information transmission is consistent with the illumination direction. Six viewing zones can be achieved by adjusting the illumination direction and updating light field information, 17 viewpoints with the density of 0.13 mm^−1^ in each viewing zone are generated. The eye-tracking technology is applied to control the backlight illumination to be in synchronization with the coded LCD panel. With eye-tracking, the direction of the information transmission can follow the position change of the viewer. As a result, 3D images can be sent to the viewer precisely, and the total viewing zone can be extended to 30 degrees. The total illumination range is determined by the structure of directional backlight; hence, in principle, the observation range can be extended further by adjusting the backlight structure. However, the viewpoints are generated by the imaging effect of LLA, and a wider illumination angle will likely to cause aberrations and degrade the 3D images quality.

### 2.2. The Optical Design of the LLA

The viewing zone parameters including the OVD, the viewing range, and the viewpoints number are predetermined according to the observation requirements. As the key component of the proposed light field display, the LLA is designed based on the predetermined viewing parameters.

**Figure 3:**
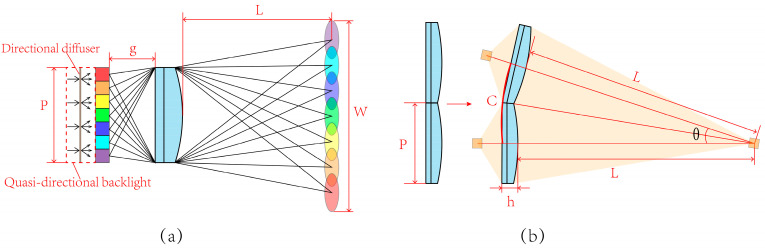
(**a**) The schematic of viewpoints generated by the lenticular lens and (**b**) the error caused by bending LLA.

As shown in Figure 3a, light rays from the quasi-directional backlight illuminate the LCD pixels. The lighted pixels are magnified by the lenticular lens and generate viewpoints at the OVD. The viewpoints number equals to the number of pixels covered by unit lenticular lens. The pitch of the LLA satisfies the relationship

(1) P=n⋅p

where P represents the pitch of the LLA, the viewpoints number in the viewing zone is denoted by n, and the pixel size of the LCD panel is denoted by p.

In Figure 3a, the distance between the LCD panel and the lens is denoted by g, and the OVD is denoted by L. According to the Gaussian imaging formula and the similarity of triangles, the focal length of the lenticular lens satisfies the relationship

(2) $$f=\frac{PL^{2}}{(P+W)L}$$

where W represents the width of the viewing zone at the OVD.

The pixels covered by the corresponding lenticular lens are magnified by the lens and superimposed at the predetermined distance to generate viewpoints. The LLA is bent to keep the same image distance of each lenticular lens, which ensures that the corresponding pixels can be magnified and superimposed precisely, as shown in Figure 3b.

It is necessary to point out that the LLA is designed to be a flat lens array. Assuming that the bending occurs at the thinnest place of the LLA, as shown in Figure 3b, the arc angle θ between two adjacent lenticular lenses can be calculated as

(3) $$\theta =2\arctan(\frac{P}{h+2L})$$

and the corresponding length of arc can be calculated as:

(4) $$C=\frac{2\pi \cdot (h+2L)\cdot \arctan(\frac{P}{h+2L})}{360^{\circ }}$$

The pitch of the LLA is 3.094 mm, and the thickness h of the LLA is 0.342 mm. Then, the calculated value of C equals to 3.09399 mm, which is close to the pitch of the LLA. The calculation indicates that the error resulted from bending the LLA is insignificant.

## 3. Verification of the Proposed Scheme

### 3.1. Light Field Pick Up and Pixel Mapping

Light field display presents spatial information and angular information simultaneously. As a reversed process, light field imaging can be considered as the coupling of spatial information and angular information. With this process, both the intensity and direction of light rays are recorded, and the spatial information and angular information are highly inter-twined [38]. The coupled information is obtained in this work with the following two steps: obtaining the light field information and mapping process to couple the spatial information and angular information.

**Figure 4:**
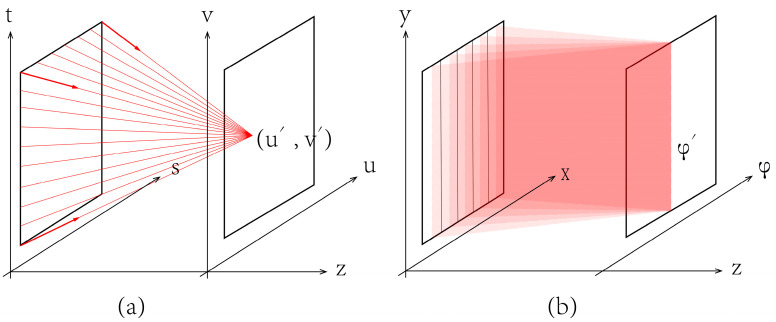
The light field parameterization model. (**a**) Full parallax; (**b**) Horizontal parallax only.

Recording the position and direction information of light rays emitted by the objects is the key to obtain the light field information. We follow a 4D light field model proposed by Levoy to parameterize the light field [6]. As show in Figure 4a, the light rays emitted from the s-t plane converge to the point on another plane u-v, and a viewpoint (u’, v’) is generated. For the light field display with horizontal and vertical parallax, cameras are placed on different coordinate on u-v plane to capture the 3D scenes on s-t plane and record the 4D light field information. The vertical parallax is regarded as unnecessary for binocular viewing. Hence, the proposed light field display neglects the vertical parallax. As shown in Figure 4b, the directional information of pitch angle is neglected, and the light information is simplified to 3D. A one-dimensional-cameras array arranged in φ direction is applied to record the light field information.

**Figure 5:**
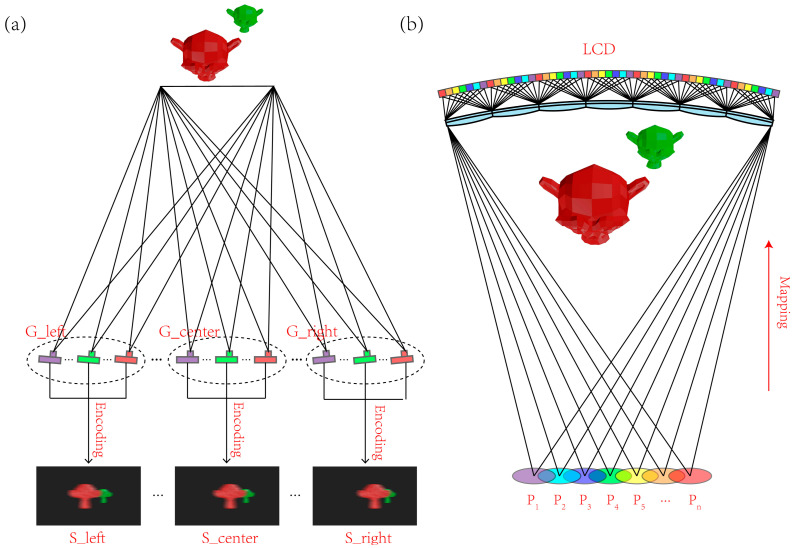
(**a**) Light field information pick-up and (**b**) the pixel mapping process.

The virtual photography method is adopted to obtain the light field information [39,40]. In the light field pick up process, a virtual-cameras array is created to shoot the 3D objects in the virtual space. As shown in Figure 5a, the cameras are divided into six groups on the basis of different viewing zones to record parallax images, and the number of cameras in each group equals the viewpoints number in the viewing zone. With this configuration, six groups of parallax images which store the intensity and direction information of the light field are obtained.

The synthesized image of each viewing zone is obtained with the encoding algorithm which executes the process of pixels mapping. The mapping relationship is shown in Figure 5b, with parallax images represented with different colors. Pixels of the parallax images are dispersed periodically, and all the parallax images in a group are synthesized to generate a single encoded image. Six groups of parallax images for different viewing zones are obtained with the virtual cameras array in a computer 3D modeling software. Each group contains seventeen parallax images.

### 3.2. Simulation

**Figure 6:**
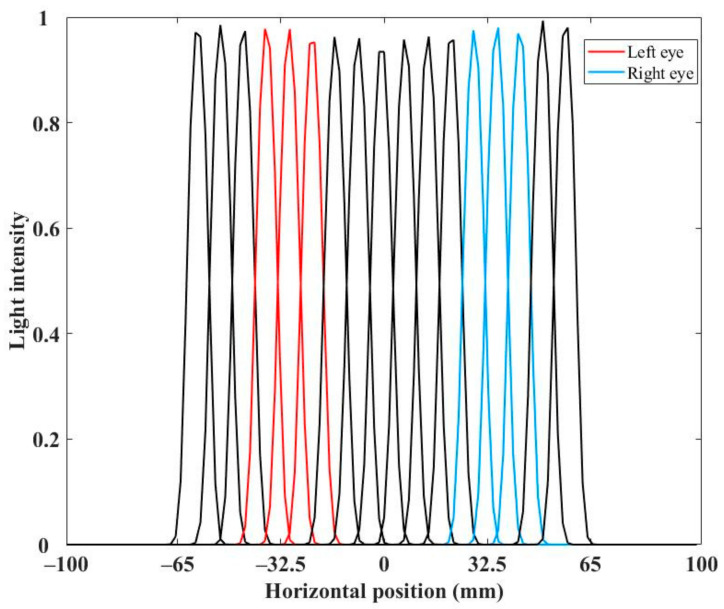
The simulated viewpoints distribution.

Simulations are carried out to verify the feasibility of the proposed light field display. An SMV light field model is built in the optical software to verify the viewpoints distribution. In the simulation model, an optical screen is placed at the OVD, and the viewpoints are obtained by measuring the optical intensity distribution on the optical screen. Figure 6 shows the measured viewpoints generated in a viewing zone.

As shown in Figure 6, the measured viewpoints are uniformly distributed in a range of 130 mm. The distance between the center of the red lines and blue lines is about 63 mm, which equals to the average human binocular distance. The red lines illustrate the viewpoints for the left eye, and the blue lines illustrate that for right eye. The intersection part between the red and blue lines is extremely small, which means that the viewpoints perceived by the left eye and right eye can be separated well, and the crosstalk between left eye and right eye is imperceptible. 

In addition, the intersection part between two adjacent viewpoints is remarkable, while the odd or even viewpoint can be separated well. With such a viewpoints arrangement, parallax between adjacent viewpoints can be smoothly shifted.

### 3.3. Experimental Results

**Figure 7:**
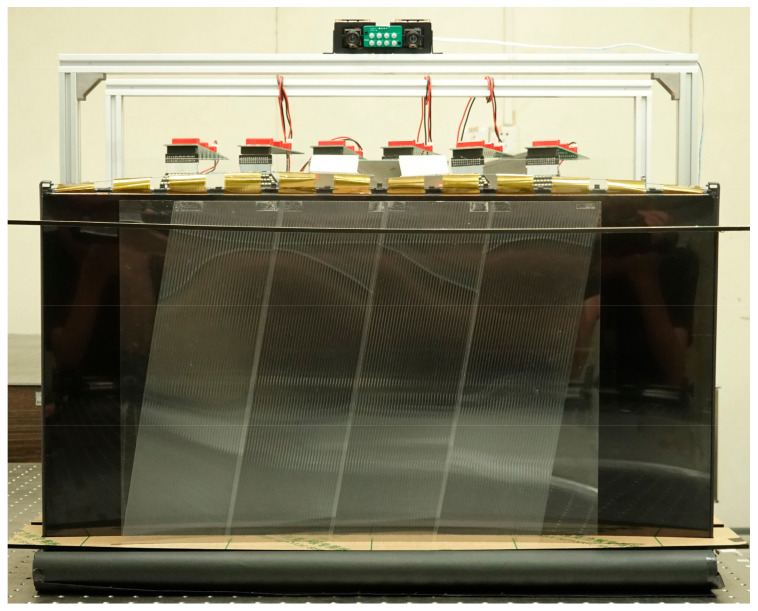
The prototype of the proposed SMV light field display.

In order to verify the feasibility of the proposed light field display, a prototype was established, and relevant experiments were conducted. The quasi-directional backlight module consists of seven units arranged side by side to achieve uniform illumination, and each unit contains a series of LEDs and LFL whose focal length is 123.4 mm. With the quasi-directional backlight, ±15-degree illumination range can be achieved. A curved LCD whose radius curvature equals to 1451 mm was placed in front of the backlight module to load the encoded light field information. The LLA was tilted 9.46-degree (arctan1/6) and placed in front of the LCD to decode the light field information and generate viewpoints. Each lens of the LLA covers 17 pixels in the horizontal direction. With such a system arrangement, the intervals of horizontal and vertical pixels in the LCD panel for constructing the same viewpoints are 17 and 6, respectively. The horizontal resolution of 3D images can be calculated as 3840 ÷ 17 = 226, and the vertical resolution can be calculated as 2160 ÷ 6 = 360. Hence, 3D images with the resolution of 226 × 360 can be perceived in each viewpoint. The detailed parameters of the prototype are listed in Table 1. Figure 7 shows the prototype of the proposed light field display system built in the experiment. 

**Figure 8:**
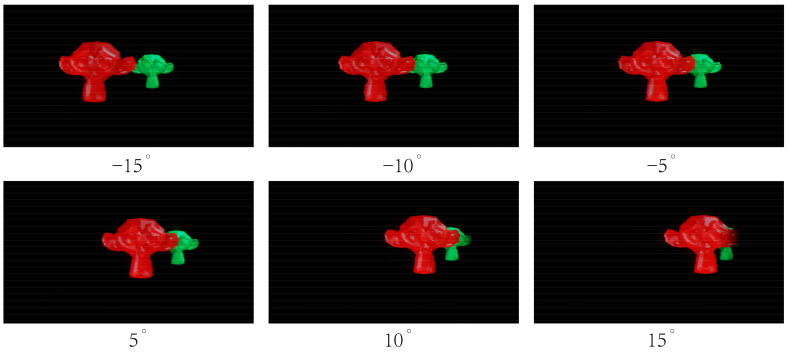
3D images generated by the prototype SMV light field display.

**Figure 9:**
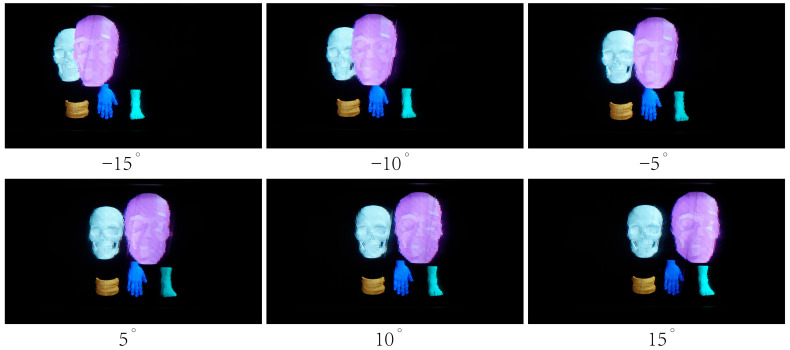
Reconstructed 3D images of human body parts.

Figure 8 and Figure 9 show the 3D images reconstructed by the prototype, and the photographs were captured by a camera placed at the OVD (1451 mm in front of the prototype). Experiment results show that vivid 3D scene can be reconstructed with the proposed light field display.

**Figure 10:**
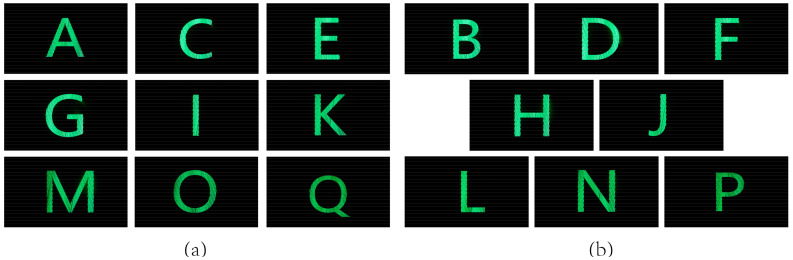
The (**a**) odd and (**b**) even viewpoints represent by different letters.

Viewpoints generated by the prototype were not measured directly, but images of different letters were encoded into a synthesized image that was then loaded on the prototype to evaluate the formation of viewpoints. Then, 17 letters from A to Q were divided into two groups according to odd and even numbers, and each group was synthesized to generate a single image for the test. In the synthesized process, letters were encoded at intervals of one viewpoint, and black images were encoded into the viewpoint between letters. A camera was placed at the OVD and moved a width of 130 mm to shoot the screen, and the experiment results are shown in Figure 10a,b.

Figure 10 shows that, when the letters were displayed at the intervals of one viewpoint, the odd or even viewpoint can be separated well, and the crosstalk is imperceptible. The experiment results are consistent with the simulation results in Figure 6. With such a viewpoints distribution, parallax between adjacent viewpoints can experience a smooth transition. The proposed light field display can provide vivid 3D images with smooth motion parallax and low crosstalk.

## 4. Discussion

The proposed scheme reduces the redundant viewpoints and improves the information utilization rate by concentrating the information to a 5-degree range. Next, 17 viewpoints are generated in the information transmission angle. The eye-tracking technique is further introduced to extend the total viewing range to 30 degrees. With this configuration, viewpoints dense up to 3.4 per degree are achieved to ensure smooth motion parallax. Furthermore, 102 viewpoints are required for a conventional light field display with a 30-degree viewing angle to achieve the same dense viewpoints, which means that 102 parallax images with the resolution of 3840 × 2160 should be utilized to generate the encoded image.

With the same dense viewpoints, the pixels count applied to synthesize each frame image in the conventional light field display is 846 × 10^6^ pixels, and approximately 20 billion (20.3 × 10^9^) pixels per second are required to process a video with 24 Hz frame rate. With the proposed scheme, the pixels count required for synthesizing each frame image decreases to 141 × 10^6^ pixels, and a 24 Hz frame rate video can be provided by processing 3.4 billion (3.38 × 10^9^) pixels per second. The total data size decreases to 16.7% of conventional light field display, and hence, the scheme proposed in this work contributes to realizing a real-time render and display of light field information.

The viewpoints in the proposed scheme are carefully arranged. With this configuration, the overlap parts of adjacent viewpoints ensure smooth motion parallax, while the overlap parts of non-adjacent viewpoints are imperceptible. Such viewpoints density reduces redundant information and suppresses crosstalk properly. As for the conventional light field display, the viewpoints density is configured casually, which results in massive redundant information and high crosstalk ratio.

**Figure 11:**
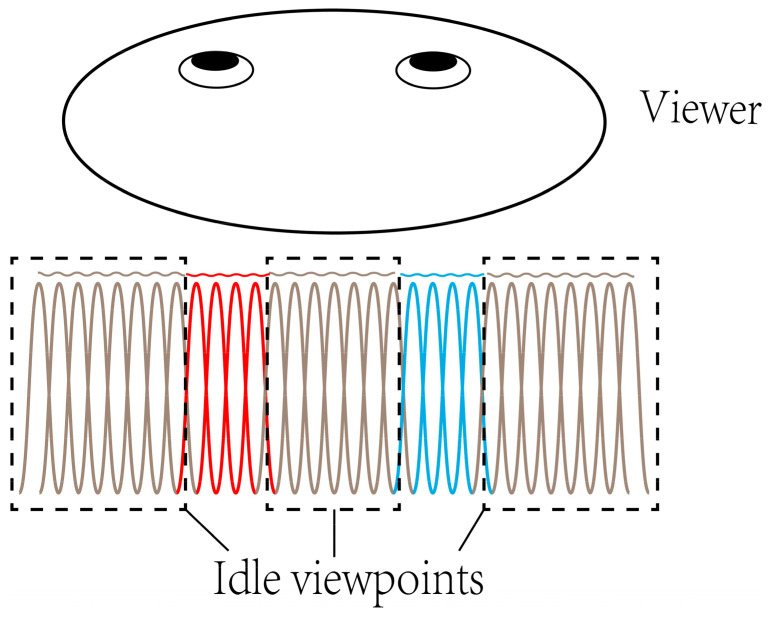
The viewpoints around eye pupils are the actually effective viewpoints.

Furthermore, the viewpoints are uniformly distributed in the viewing zone whose width is set to the twice of the average human binocular distance in the proposed scheme. However, the effective viewpoints are the parts that are situated in the periphery of pupils. As shown in Figure 11, the red lines illustrate the viewpoints for the left eye, the blue lines illustrate those for right eye, and the others are idle. Since the actually effective viewpoints are those around eye pupils, the quality of light field display can be further improved, and the data size can be further reduced by reducing the idled viewpoints. It is possible to achieve the improved scheme by further concentrating the viewing area into the human eye and combining with time-multiplexing technique to switch the information for the left and right eye [41].

The backlight module contains seven units, the total number of columns is 68 in each of the seven units, and each column consists of 21 LEDs. The directional autostereoscopic display system proposed in our previous work [42] normally lightens seven columns LEDs together in each unit to ensure the illumination width and provide homogeneous illumination over the entire screen for one eye. Since the illumination range is re-modulated by the LLA, each unit requires only one column LEDs (21 × 7 = 147 LEDs with a total power 3.5 W) with the same parameters for the proposed SMV light field display to provide the entire homogeneous illumination for a pair of eyes. Thus, an illumination power of 3.5 watt is sufficient to lighten a 31.5-inch light field display, which takes up only 1.5% of the illumination power required for planar display of similar configuration. Detailed power consumptions of backlight module are shown in Table 2.

A multi-user directional backlight autostereoscopic display was achieved in our previous work [43]. In principle, the SMV light field display presented in this work can be easily improved into a multi-user one with the technique similar to the previous work. The backlight module in the SMV light field system can be controlled to generate a multi illumination area. While the difference is that light field information varies with horizontal viewing position, LCD panel refreshing is required to update the light field information for different viewing zones. Since the information transmission is always consistent with the illumination direction, multi-viewing zones can be achieved by synchronously adjusting the directional backlight to switch the illumination direction and refreshing LCD panel to update light field information. With this multi-user scheme, an LCD panel with 60 Hz refresh rate can achieve two 30 Hz viewing zones, and the multi-viewing zone can be achieved at the cost of refreshing rate. Hence, an LCD panel with a high refresh rate is required to improve the display effect.

**Figure 12:**
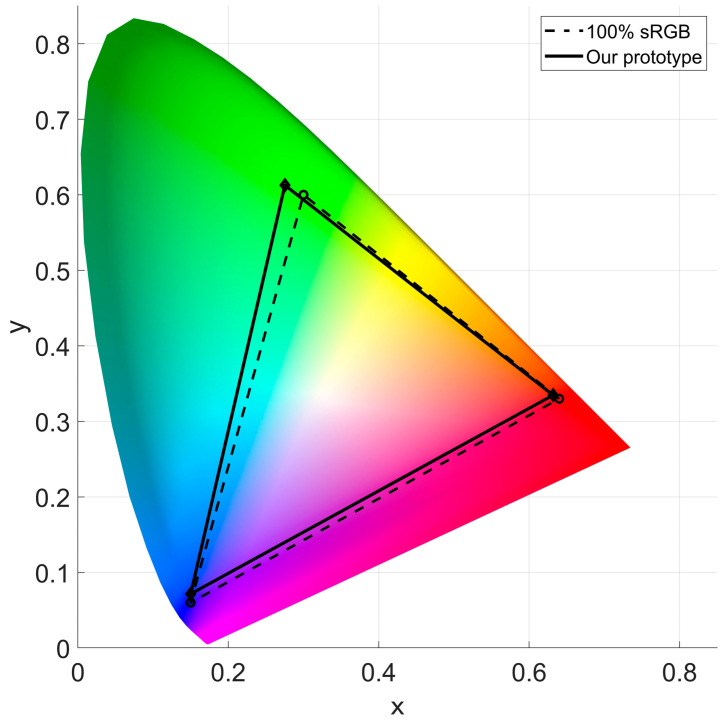
Color gamut coverage comparison of 100% sRGB and the prototype SMV light field display.

The color gamut of display shows the range of all colors that can be represented or reproduced by a display system. Table 3 shows the measured color gamut coordinates value of the prototype SMV light field display system. Figure 12 shows the color gamut coverage of the prototype, the coverage of 100% sRGB is also presented as a contrast. The measured results indicate that the color gamut coverage of the prototype equals to 101.65% sRGB, which shows excellent color performance ability.

The proposed SMV light field display reduces the pressure of data processing and achieves high energy efficiency, providing a new way to realize high quality light field display. The proper viewpoints configuration ensures smooth motion parallax and extremely low crosstalk. Ultimately, 3D images with excellent color performance have been achieved. However, the edge of reconstructed 3D images shows marked serration, and the complex system arrangement leads to a rather large thickness compared with a conventional 2D display. The image processing technique may help reduce jagging, and the optimization of optical structure may further compact the system.

## 5. Conclusions

Here, an SMV light field display is proposed to concentrate the information in the viewing zone of a single user by reducing the redundant viewpoints. The quasi-directional backlight is proposed, and an LLA with long focal length is applied to achieve the viewing zone, in which 3.4 viewpoints per degree are generated to ensure parallax saltation-free. The total horizontal observation area can be extended to 30 degrees or above by further introducing the eye-tracking technique. The prototype built in our experiment confirms that vivid 3D images with correct geometric occlusion and smooth motion parallax can be obtained.

With the system configuration, the illumination range is concentrated in the viewing zone of a single user, and all pixels of the LCD panel are concentrated in the viewing zone to generate viewpoints. The information utilization rate and the energy efficiency are substantially improved. By reducing redundant viewpoints, the total data size reduces to 16.7%. Hence, the requirements of information transmission bandwidth are reduced, which contributes to realizing a real-time render and display of light field information. This study further demonstrates that only a fraction of the illumination power of a planar display is required for directional light field display, making this display technology particularly attractive for 3D display.

In summary, an energy-efficient SMV light field with high information utilization has been proposed. The information is highly concentrated in the viewing zone to generate viewpoints. The viewpoints density is properly arranged to ensure smooth motion parallax and to achieve low crosstalk ratio. Meanwhile, the illumination is also concentrated in the viewing zone, which ensures less energy to achieve enough brightness and makes the proposed light field display energy efficient. Furthermore, the eye-tracking technique is introduced to extend the total observation area and improve the practicability of the proposed light field display. High quality 3D images with extreme color performance have been achieved.

The proposed light field display featuring high information utilization and energy efficiency is of great significance to realize 3D display with more efficient information and energy utilization. Further studies focusing on thickness reduction may expend the practical applications, and we expect that it can be potentially applied in a head-up display.

## Figures and Tables

**Table 1:** Specifications of the Proposed System.

Item	Configuration	Detail
The directional backlight module	Number of V-shape backlight unit	7
	Focal length of LFL	123.4 mm
	Pitch of LFL	80 mm
	Thickness of LFL	2 mm
	Thickness of backlight module	133.98 mm
	Curvature radius of backlight module	1500 mm
The LCD panel	Size of the screen	31.5-inch
	Resolution	3840 × 2160
	Pixel size	0.182 mm
	Curvature radius of LCD panel	1451 mm
The LLA	Focal length of LLA	34.87 mm
	Pitch of LLA	3.094 mm
	Thickness of LLA	0.342 mm
	Tilt angle	9.46°
	Number of covered pixels	17
	Gap between LLA and LCD	35.7 mm

**Table 2:** The power consumption of backlight module.

Item	Value
Units number	7
Power consumption of each LED	0.024 W
The number of LEDs in each column	21
Power consumption of a column LEDs	0.504 W
Power consumption of light field mode	3.5 W
Power consumption of planar display mode	239.9 W

**Table 3:** Color gamut coordinates of the prototype.

Color	x	y
R	0.6319	0.3347
G	0.2753	0.6127
B	0.1501	0.0715

## Data Availability

Data is contained within the article.
